# Eco-friendly method for construction of superhydrophobic graphene-based coating on copper substrate and its corrosion resistance performance

**DOI:** 10.1038/s41598-022-22915-5

**Published:** 2022-10-26

**Authors:** D. M. Ragheb, A. M. Abdel-Gaber, F. M. Mahgoub, M. E. Mohamed

**Affiliations:** 1 Chemistry Department, Faculty of Science, Alexandria University, Alexandria, Egypt; 2 Materials Science Department, Institute of Graduate Studies & Research, Alexandria University, Alexandria, Egypt

**Keywords:** Materials chemistry, Surface chemistry

## Abstract

In this work, Ni and Ni-graphene, Ni-G, films were electrodeposited on copper substrate by potentiostatic deposition. To achieve superhydrophobicity, myristic acid, MA, was used to modify the surface of the electrodeposited coatings. The manufactured Ni film modified with myristic acid, Ni-MA, and the Ni-G film modified with myristic acid, Ni-G-MA, show excellent superhydrophobic, SHP, properties with a water contact angle of 159° and 162°, respectively. The surface morphology of the prepared SHP films was investigated using a Scanning Electron Microscope, and the results revealed micro-nano structures in both Ni-MA and Ni-G-MA films. The Fourier Transform Infrared Spectrophotometer data showed that the Ni-MA and Ni-G-MA films were successfully grafted on the copper metal. The Ni-G-MA film possessed higher chemical stability and mechanical abrasion resistance than Ni-MA. The Ni-MA and Ni-G-MA films exhibit long-term durability in the outdoor environment for more than four months. The potentiodynamic polarization and electrochemical impedance spectroscopy results demonstrated that the SHP films on the copper substrate exhibit remarkable corrosion resistance in 0.5 M NaCl.

## Introduction

Surfaces with a high contact angle (CA) larger than 150° and a low sliding angle (SA) less than 10° are referred to as superhydrophobic, SHP. The SHP surface manufacturing principles are based on natural phenomena, including lotus, rose, and rice leaves^1,2^. Because of its relevance, such as drag reduction^3^, corrosion resistance^4^, self-cleaning capabilities^5^, water/oil separation^6^, and so on, SHP surfaces have recently sparked significant interest in academic research and prospective industrial applications. Two elements must be present to construct a superhydrophobic surface: rough surface texture with a distinct binary structure and surface chemistry modification with a low-free-energy coating^7^. Chemical vapour deposition^7^, sol–gel^8^, chemical etching^9^, spray^10^, and electrodeposition^11^ have all been presented as ways to generate bio-inspired SHP surfaces by changing surface morphology and chemical compositions. However, most of these approaches are limited by the need for specialized equipment or difficult process control^12^. On the other hand, electrodeposition is a simple way to produce SHP coatings on metals, metal oxides, and conducting polymers^13,14^. Electrodeposition has become a competitive method to produce superhydrophobic surfaces owing to its advantages, including scalability, ease of control, simplicity, low cost, and fabrication of a robust SHP coating^13,15–18^.

The prepared SHP coating has some defects, including low mechanical stability, vulnerability to external damage, and rapid superhydrophobicity loss. Additionally, SHP surfaces are prone to break down in specific situations (for instance, corrosive ions, alkali, and acid), limiting their protective action against metal corrosion^19^. Numerous approaches were used to attain low-energy surfaces employing biological poisons such as fluoroalkyl silane (FAS)^20^. As a result, there is still a pressing need to produce chemically and mechanically stable SHP coatings using simple and environmentally safe approaches.

Copper is an important engineering element with many industrial uses, including electrical power lines, heat conductors, and water supply pipelines^21–25^. In an environment containing chloride ions, the copper substance will rapidly corrode. According to many authors, minimizing the copper substrate's contact surface with corrosive liquids would significantly increase corrosion resistance^26–29^. As a result, developing SHP coating for copper is critical to strengthening corrosion resistance. Nickel is a main industrial metal with desirable qualities such as hardness, magnetism, and corrosion resistance. As nickel is applied to copper, the deposited nickel reduces bare copper corrosion. The deposited nickel coating can offer unique advantages, such as self-cleaning and superior corrosion resistance when combined with superhydrophobicity^30^. Graphene is a honeycomb-like carbon allotrope with a two-dimensional structure^31^. One of the most astonishing nanomaterials is graphene, which is the thinnest and one of the most durable carbon-based structures. Because of its strength, single atomic layer thickness, chemical inertness, hydrophobicity, impermeability to most gases, and preventing metal oxidation at the expense of its oxidation, graphene is a desirable material for coatings, notably anti-corrosion coatings^32,33^. Carbon-based films often exhibit weak substrate bonding and ineffective hydrophobicity, which greatly restricts their applicability^34^. Fortunately, doping with metals, such as nickel, or non-metals, improved substrate homogeneity and adherence^35^.

This study aims to use an electrodeposition approach to create Ni film and Ni film doped with graphene on a copper substrate. Then the films were modified with myristic acid, an eco-friendly low surface energy compound, resulting in SHP surfaces. The surface topography and chemical composition of the produced SHP surfaces were studied using a Scanning Electron Microscope, SEM, and a Fourier Transform Infrared Spectrophotometer, FTIR, respectively. The produced SHP films were tested for wettability, durability in outdoor environment, chemical and mechanical stability, and corrosion performance in an aqueous solution of 0.5 M NaCl.

## Experimental

### Materials

A copper plate with dimensions of (2.0 cm × 1.0 cm × 0.3 cm) is utilized as the working electrode. Sodium hydroxide, nickel chloride hexahydrate, nickel sulphate, sulfuric acid, boric acid, anhydrous ethanol, and myristic acid of analytical quality were utilized.

### Sample preparation

Before electrodeposition, the copper substrate was polished using several classes of SiC paper, starting with the sieve (grade 150) and going to the finest (800 grade). The copper substrate was submerged for 10 min in a soap solution, followed by 1 min in 0.5 M H_2_SO_4_. Before being submerged in the electrodeposition bath, the copper substrate was washed with distilled water. Nickel ion sources NiSO_4_ (176 gL^−1^), and NiCl_2_.6H_2_O (40 gL^−1^), as well as the buffering agent H_3_BO_3_ (60 gL^−1^), make up the electrodeposition path. A platinum rod was used as an anode, and a copper substrate was employed as the cathode to produce a nickel film, with 1.0 cm between them and potential of 8.75 V (optimum potential). A graphite rod was utilized as an anode with a potential of 10.0 V (optimum potential) to create nickel graphene film, Ni-G. Anodic electrochemical exfoliation was used to create graphene, which includes employing a positive current to remove electrons from graphite, the anode, producing a positive charge. The positive charges promote the intercalation of Cl^-^ negative ions into graphite. According to reports, water is crucial to the electrochemical process because it can generate oxygen and hydroxyl radicals that can help with the exfoliation and intercalation of graphite^36^. These lead to an increased interlayer space and eventual exfoliation of the graphene sheets. The prepared Ni and Ni-G films were cleaned with distilled water after electrodeposition and dried for 24 h at room temperature. Dry films were soaked for 15 min in 0.01 M myristic acid, then washed with ethanol to remove the physically stacked myristic acid molecules on the sample surface, and dried at room temperature. The Ni-MA film, Ni film modified by myristic acid, and the Ni-G-MA film, Ni-G film modified by myristic acid, were subjected to numerous evaluations and characterization procedures.

### Surface analysis

The surface topography of electrodeposited coating was examined by SEM (JEOL, model JSM-200 IT). The chemical composition of the produced SHP coating was investigated using FTIR (model Bruker Tensor 37). The spectra were collected between 4000 and 400 cm^−1^. The CA and SA were measured with 5 µl of water droplets using an optical contact angle goniometer (Rame-hart CA instrument, model 190-F2). The averages of three measurements taken at distinct substrate sites were used to calculate CA and SA.

### Mechanical abrasion resistance

The mechanical characteristics of the produced SHP films were investigated using the scratch, tape peeling, and sand impact tests. The scratch test was performed by applying the SHP film to a SiC paper (1200 grade) that operated as an abrasion surface. The SHP film was subjected to a 1.5 kPa pressure. The CA of a water droplet on the prepared SHP films was evaluated for every 50 mm of abrasion. The tape peeling test was done by applying strong adhesive tape on the SHP coat and then using a finger to peel the tape off. Repetitive tape application and peel-off cycles were utilized to evaluate the mechanical stability of the SHP coating. For the sand impact test, 50 g of sand in a funnel was dropped from a height of 50 cm to strike the SHP coated copper. The sample's water superhydrophobicity was tested by measuring the CAs and SAs for every 50 g of sand that hit the SHP surface.

### Chemical stability

Different samples of the produced SHP films were immersed for one hour in solutions of different pH (pH = 1–13), then the CA and SA were determined at each pH. The solution pH was modified by sulphuric acid (0.5 M) and sodium hydroxide (0.5 M).

### Corrosion test

The corrosion-resistant performance of coated copper was measured by electrochemical impedance spectroscopy (EIS) and potentiodynamic polarization measurements in 0.5 M NaCl aqueous solution. ACM potentiostat/frequency response analyzer (UK) was used to perform EIS and potentiodynamic polarization studies utilizing a three-electrode cell. The counter electrode is made of platinum, and the reference electrode is made of Ag/AgCl. Working electrodes are bare and coated copper with Ni, Ni-G, Ni-MA, and Ni-G-MA. The working electrodes were covered with an epoxy coating, leaving 1 cm^2^ exposed and in contact with the test solution. The working electrode was introduced into the cell containing 0.5 M NaCl solution at room temperature (25 °C). The test solution was left for 20 min before electrochemical tests to determine the open circuit potential. The frequency range employed for the EIS experiments was 0.01 ≤ f ≤ 3.0 × 10^4^ Hz, with an applied potential signal amplitude of 10 mV around the open circuit potential. The polarization measurements were taken at a 30 mV min^−1^ scan rate, starting at cathodic potential (E_rest_ − 250 mV) and progressing to anodic potential (E_rest_ + 250 mV). Experiments were double-checked to ensure the measurements were accurate, and the results were within 2% error.

## Results and discussion

### Morphology and wettability results

**Figure 1:**
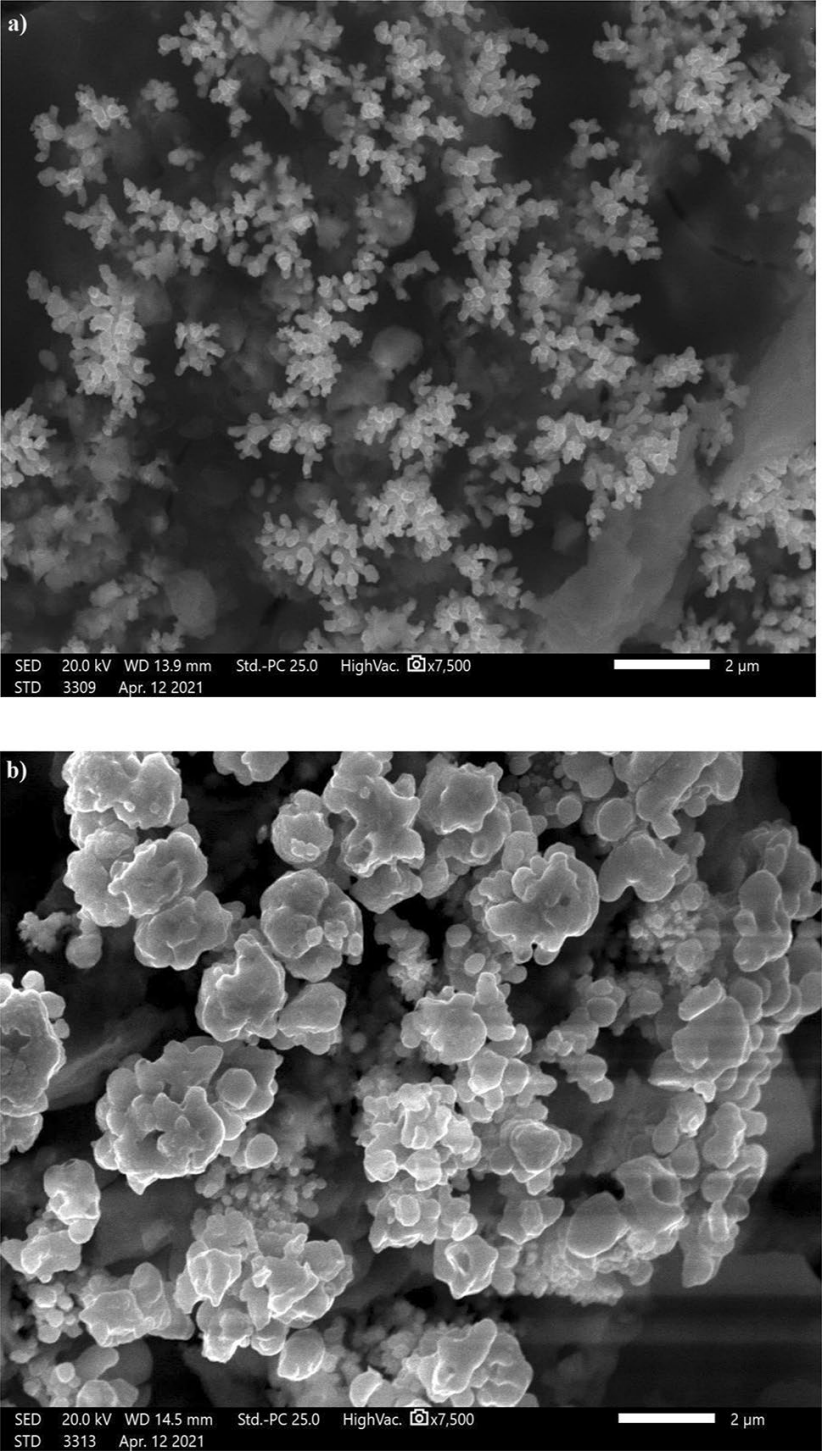
SEM images of copper grafted by (**a**) Ni-MA and (**b**) Ni-G-MA.

**Figure 2:**
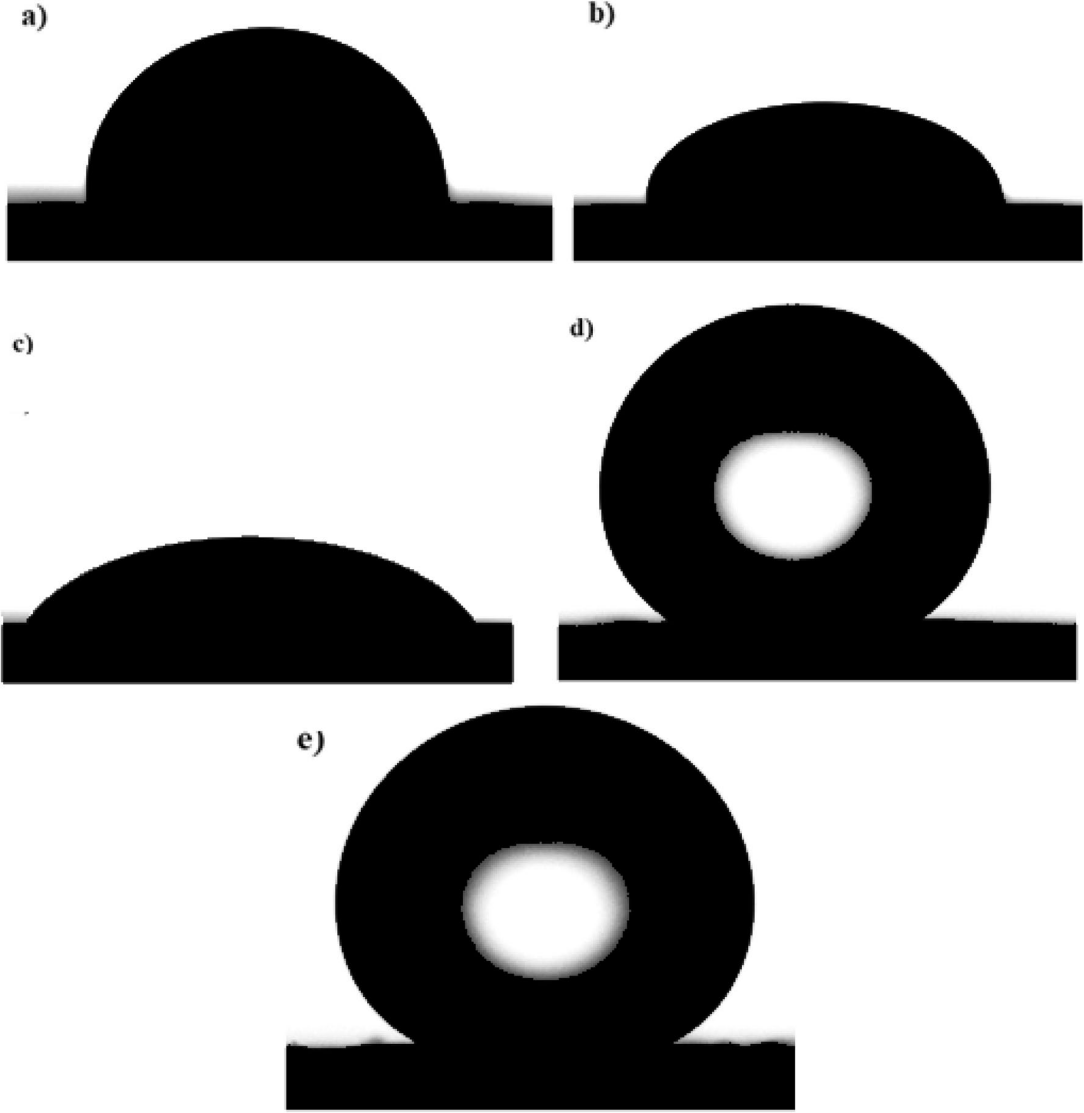
Wettability micrographs of (**a**) bare copper and coated copper by (**b**) Ni, (**c**) Ni-G, (**d**) Ni-MA, and (**e**) Ni-G-MA.

Since surface topography is a key factor in SHP characteristics, scanning electron microscopy was utilized to investigate the surface topography of the produced SHP films. Figure 1a displays the SEM image of copper coated by Ni-MA; it is obvious that the surface has micro-nano structures of dendritic shape. Figure 1b shows the SEM image of copper coated with Ni-G-M, the surface features micro-nano structures in the shape of cauliflowers. Graphene could serve as a nucleation site to improve the nucleation rate, so it improves surface roughness, which is the primary requirement for superhydrophobicity^31^. To assess the wettability of the bare and coated copper by Ni, Ni-G, Ni-MA, and Ni-G-MA, the CAs were evaluated. The bare copper and the coated copper by Ni and Ni-G have CAs of 58°, 24°, and 12°, respectively, but the water droplet does not slide on them. It is obvious that coating the copper with a rough deposit of Ni or Ni-G enhances the hydrophilicity of the bare copper, which has high surface energy. It is reported that the low surface energy rough surfaces are typically superhydrophobic, whereas high surface energy rough surfaces are typically superhydrophilic^37^. The Ni-MA and Ni-G-MA films exhibit CAs of 159° and 162° and SAs of 1° and 3°, respectively. The shape of a water droplet on the bare and coated copper is revealed in Fig. 2. These findings suggest that doping Ni films with graphene increase the films' superhydrophobicity. This means that the higher adherence of the myristic acid to the rough structure of the Ni-G film than that of the Ni film is the cause of the higher superhydrophobicity of the Ni-G-MA film. According to the Cassie–Baxter theory, the Ni-G-MA film stores more air in its micro-nano structures^38^.

### FTIR results

**Figure 3:**
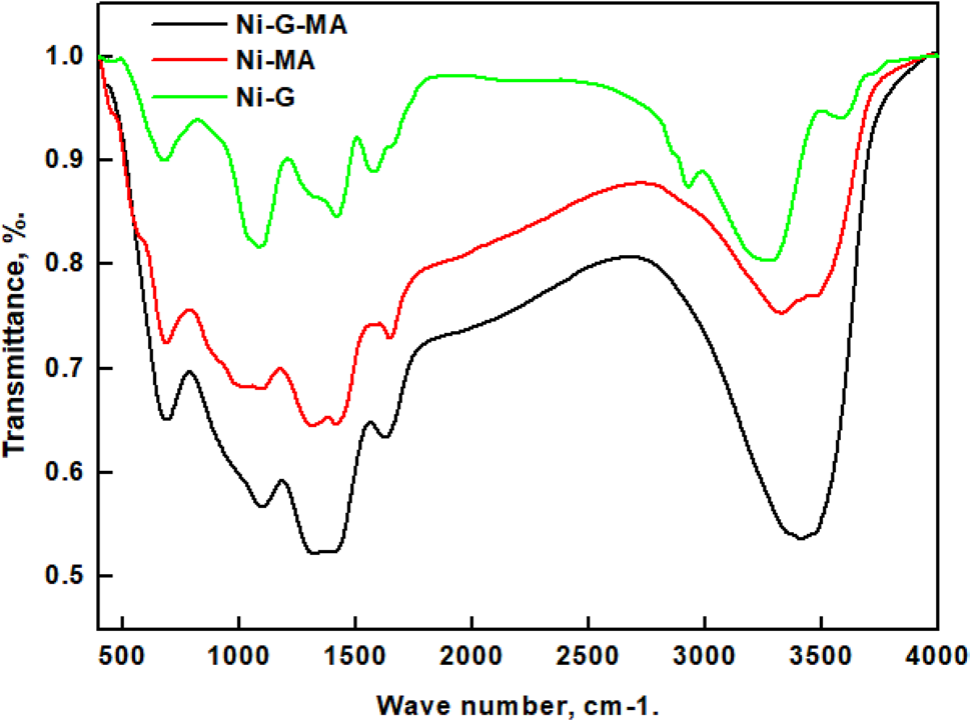
FTIR spectra of copper grafted by Ni-G, Ni-G-MA, and Ni-MA.

The FTIR spectra of copper coated with Ni-G, Ni-MA, and Ni-G-MA are presented in Fig. 3. The spectrum for copper grafted by Ni-G demonstrates the characteristic peaks of graphene. Peaks at 3330 cm^−1^ and 1620 cm^−1^ are ascribed to stretching and bending vibrations in O–H bonds, respectively^39^. The –CH–, –CH_2_–, and –CH_3_– asymmetry and symmetry vibrations are ascribed to the two peaks at 2936 cm^−1^ and 2830 cm^−1^^40,41^. The peak at 1640 cm^−1^ represents the C=C double bonds in the polycyclic aromatic graphene ring. The C–O–C band typical of epoxy emerges at 1060 cm^−1^^42^. The peak at 692 cm^−1^ is attributed to Ni(OH)^43^. The spectrum for copper grafted by Ni-MA depicts a peak at 1655 cm^−1^ assigned to the coordinated COO functional group of myristic acid. The O–H bond tension vibration is represented by the band at 3440 cm^−1^^39^. The two peaks at 1419 cm^−1^ and 1313 cm^−1^ are attributed to the C-H bending vibration^44^. The peaks of copper coated by Ni-G-MA are the same as those of copper coated by Ni-MA and also show the characteristic peaks of graphene, confirming that the Ni-G film is grafted by myristic acid. In the case of copper-coated by Ni-G-MA, the higher peak intensity shows that a higher concentration of myristic acid is adsorbed to the deposited film, resulting in a film with a higher superhydrophobicity^45^.

### Mechanical abrasion resistance

**Figure 4:**
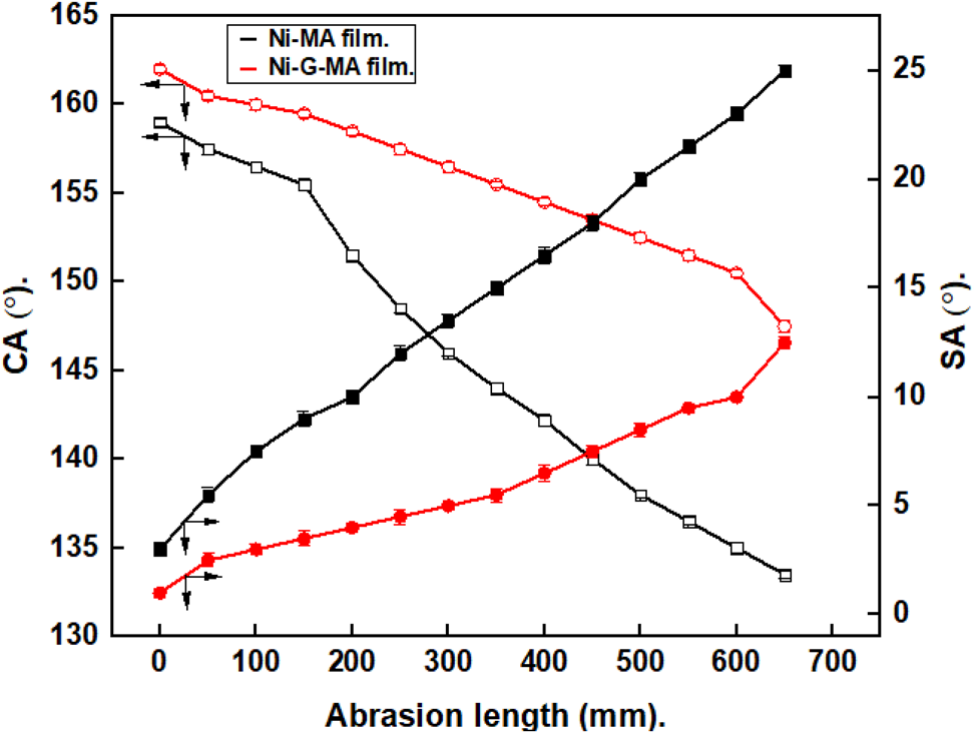
Variation of CAs and SAs with the abrasion length for copper grafted by Ni-MA film and Ni-G-MA film.

The SHP materials' rough surface is sensitive to mechanical wear. SHP coatings' resistance to abrasion has become a crucial aspect of their industrial uses. The resistance of the generated SHP films to mechanical abrasion was evaluated using scratch, tape peeling, and sand impact tests. According to Fig. 4, the abrasion length affects the CAs and SAs of the water droplets on the produced SHP films. As the abrasion length increased, the contact angle dropped while the sliding angle rose, as seen in the Figure. The prepared SHP Ni-MA film exhibits superhydrophobicity up to a 200 mm abrasion length. In comparison, the prepared SHP Ni-G-MA film retains its superhydrophobicity up to a 600 mm abrasion length. These findings demonstrate that doping the Ni-MA SHP film with graphene manufacturing Ni-G-MA enhances its mechanical stability. The abrasion of the SHP surfaces leads to destroying the micro-nano structures on the surface, and so the surface roughness is decreased. So, the Cassie-Baxter state of the SHP surfaces is converted into Wenzel's state. As a result, the water droplet and surface become locked together, and their adhesiveness increases, which causes the superhydrophobic properties to diminish, the CA decreases, and the SA increases^16,46^. The prepared SHP Ni-G-MA has more abrasion resistance than many of the reported values^47,48^.

**Figure 5:**
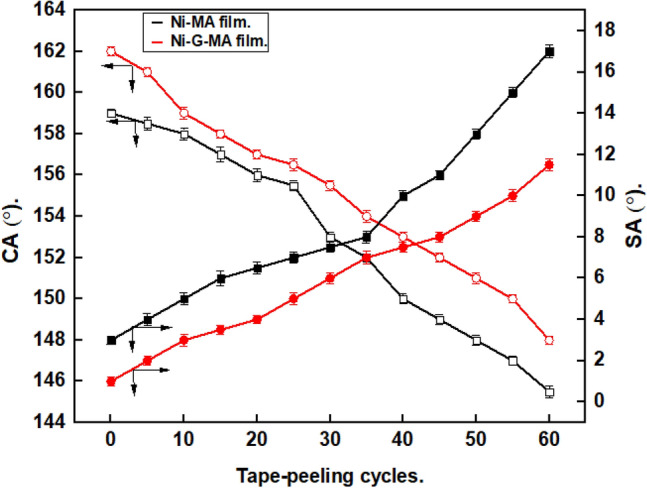
Effect of cyclic tape peeling on the CAs and SAs of copper grafted by Ni-MA film and Ni-G-MA film.

Figure 5 shows the effect of cyclic tape peeling on the CAs and SAs of the SHP films. The cyclic tape peeling test had little impact on the surface's superhydrophobicity because the CA reduced and the SAs rise slowly throughout the test. As shown in the Figure, the prepared SHP Ni-MA film exhibits superhydrophobicity until 40 cyclic tape peeling. While the prepared SHP Ni-G-MA film retains its superhydrophobicity up to 65 cyclic tape peeling, indicating strong binding between the myristic acid and the deposited rough Ni-G coat. The prepared SHP Ni-G-MA has resistance to a cyclic tape peeling greater than many of the reported values^18,49,50^.

**Figure 6:**
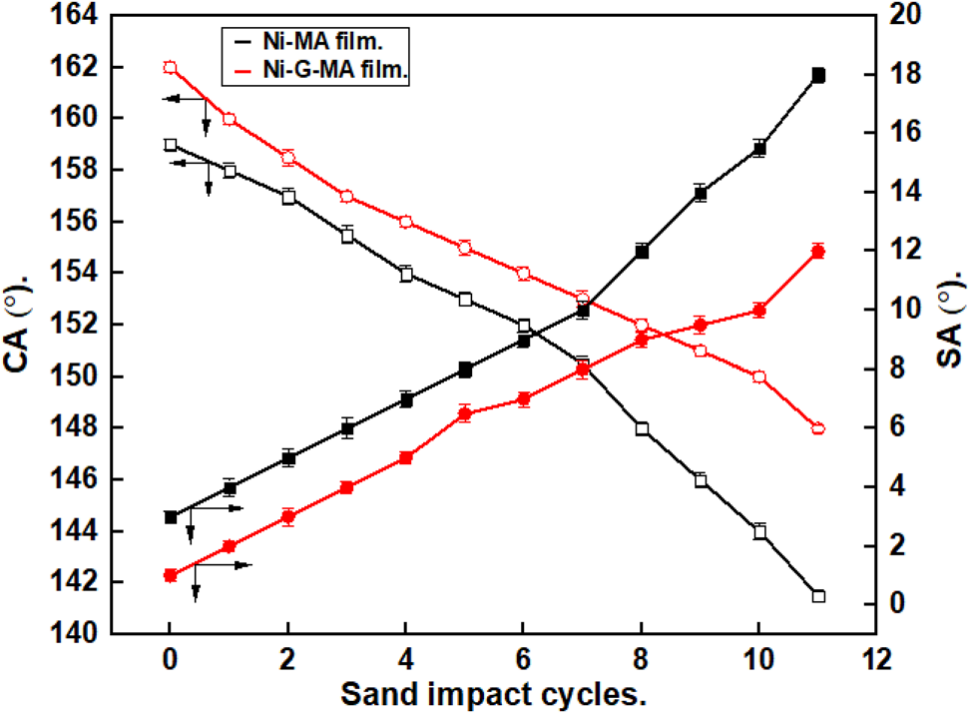
The sand impact on the CAs and SAs of copper grafted by Ni-MA film and Ni-G-MA film.

Sand abrasion tests were carried out to assess the mechanical performance of the SHP coatings, Fig. 6. The prepared SHP Ni-MA film exhibits superhydrophobicity until 7 sand impact cyclic. In comparison, the prepared SHP Ni-G-MA film retains its superhydrophobicity up to 10 sand impact cyclic. The prepared SHP Ni-G-MA has resistance to sand impact higher than many reported values^51,52^.

The durability of the SHP sample is further tested by storage in an ambient atmosphere. After 4 months of storage in air, the values of the CAs of Ni-MA and Ni-G-MA are 152° and 155°, and the SAs are 8° and 6°, respectively. These results indicate that the prepared SHP films are stable in air and thus exhibit long-term stability and durability.

### Chemical stability

**Figure 7:**
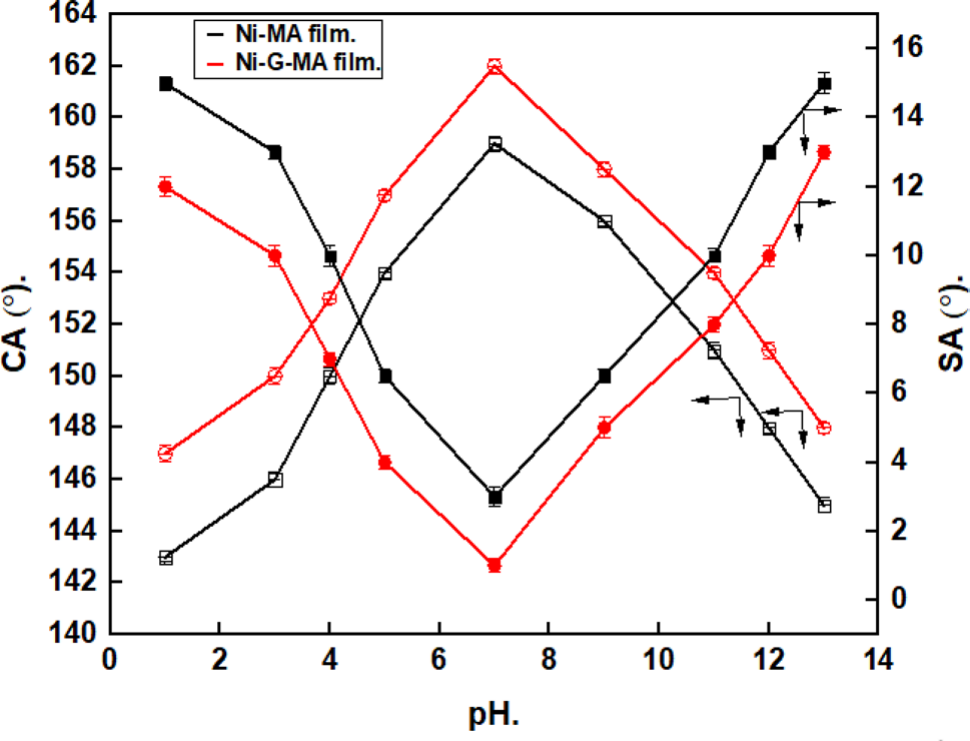
Variation of water droplets' pH and their CAs and SAs on the copper coated by Ni-MA film and Ni-G-MA film.

A chemical stability test needs to be carried out to confirm that the developed SHP films may be applied in industrial applications. Different samples of the copper grafted by SHP films were immersed in aqueous solutions of pH from 1 to 13 for one hour, then the CAs and SAs were estimated at each pH. The CAs and SAs of water droplets on copper grafted by SHP films along with pH, are shown in Fig. 7. In the pH range of 4–11, the Ni-MA film remains SHP. In the pH range of 3–12, the Ni–G–MA film exhibits superhydrophobicity, with contact angles that are frequently higher than 150° and sliding angles that are less than 10°. The SHP Ni-MA film's chemical stability in both acidic and basic conditions was improved due to the presence of graphene. Low surface energy and surface roughness are two critical requirements for superhydrophobic film fabrication. As a result, aggressive acidic and basic liquids can lower the density of hydrophobic groups on the surface and destroy the surface's micro/nanostructures, causing the surface to lose its superhydrophobic properties. So, the water contact angle will be decreased, and the water sliding angle will be increased^16,53–55^. The SHP-coated copper by Ni-G-MA has greater chemical stability than several previously recorded values^56,57^.

### Corrosion resistance test

#### Potentiodynamic polarization results

**Figure 8:**
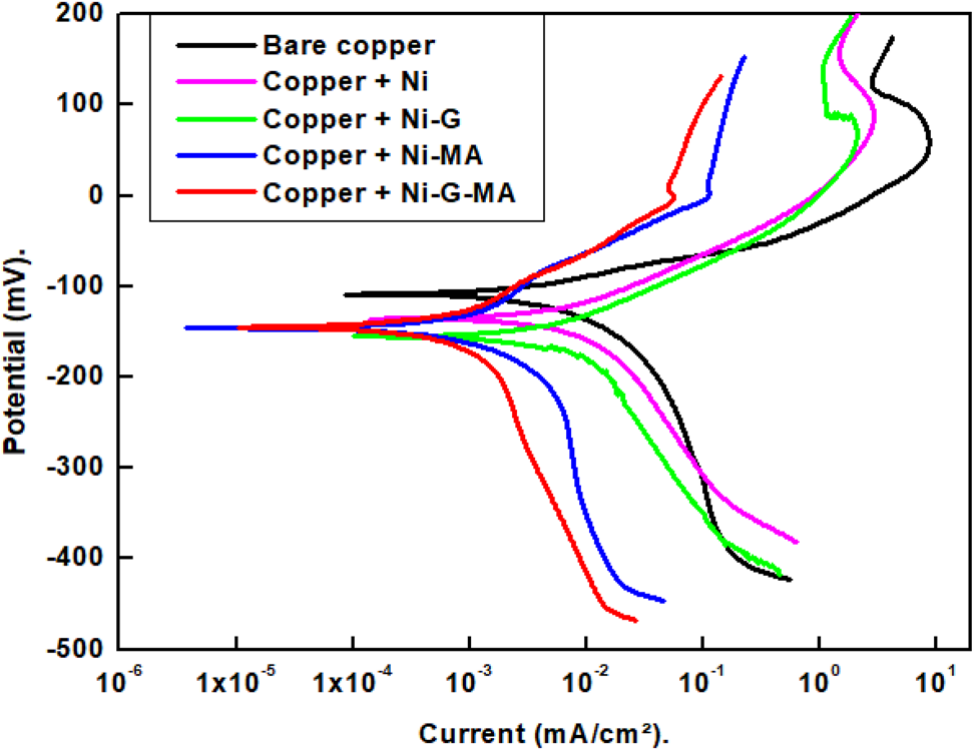
Potentiodynamic polarization curves for bare and coated copper with Ni, Ni-G, Ni-MA, and Ni-G-MA in 0.5 M NaCl solution.

The corrosion resistance of bare and coated copper by Ni, Ni-G, Ni-MA, and Ni-G-MA was examined using the potentiodynamic polarization technique. The potentiodynamic polarization diagrams of uncoated and coated copper in a 0.5 M NaCl solution are depicted in Fig. 8. According to Eq. (1), the cathodic polarization graphs clearly show that a limiting diffusion current, I_L_, is caused by the oxygen reduction^58,59^.

1 $$ {\text{O}}_{{2}} + {\text{2H}}_{{2}} {\text{O }} + {\text{ 4e}} \to {\text{4OH}}^{ - } $$

As a result, mass transfer regulates the cathodic process. The anodic polarization curve shows a passivity behavior. This is due to the rapid accumulation of corrosion products in the case of bare copper or the formation of a passive layer in the case of coated copper that has been manufactured^58,59^. The potentiodynamic polarization parameters include corrosion potential, E_corr_., corrosion current density, i_corr_., the anodic Tafel slope, β_a,_ the cathodic Tafel slope, β_c,_ and protection efficiency, %P, of the bare and the coated copper are summarized in Table 1. Equation (2) is used to calculate the protection efficiency %P^60^:

2 $$ \% {\text{P}} = \, \left[ {\left( {{\text{i}}^{{\text{o}}}_{{{\text{corr}}{.}}} - {\text{ i}}_{{{\text{corr}}{.}}} } \right)/{\text{ i}}^{{\text{o}}}_{{{\text{corr}}{.}}} } \right] \, \times { 1}00 $$

where i^o^_corr_. and i_corr._ are the corrosion current density for bare and coated copper. The i_corr._ values for copper coated with Ni-MA (1.06 µA cm^−2^) in 0.5 M NaCl solution at ambient temperature are clearly lower than those for bare copper (12.91 µA cm^−2^). This could be owing to the coated copper’s SHP character, where air trapped in micro-nano structures reduces the contact area between the prepared SHP coated copper and the aqueous solution, lowering the i_corr_^61^. For SHP copper coated with Ni-G-MA, we observed a further decrease in i_corr_ value (0.93 µA cm^−2^), which could be due to the presence of graphene. The i_corr._ values for copper coated with Ni and Ni-G in 0.5 M NaCl solution are 5.01 µA cm^−2^ and 3.79 µA cm^−2^ which are high values corresponding to that of the SHP coated copper, and this reflects the role of the superhydrophobic layer in decreasing the corrosion rate. The coated copper shows a cathodic shift in the potential where all coated copper samples have potentials that are more negative than bare copper in a solution of 0.5 M NaCl which corresponds to the dominant cathodic reaction control^62,63^. Higher values of βc as compared with βa confirm the dominant control in the cathodic reaction^64^. The higher corrosion resistance of copper coated with Ni-G-MA film is due to the same factors described before to demonstrate Ni-G-SA films’ superior chemical and mechanical stability. So, the prepared copper coated with Ni-G-MA has greater protective efficiency than Ni-MA.

**Table 1:** Potentiodynamic polarization parameters for bare copper and coated copper by Ni, Ni-G, Ni-MA, and Ni-G-MA in 0.5 M NaCl solution.

Deposit	− E_corr_ (mV)	β_a_	− β_c_	i_corr_ (µA cm^−2^)	%P
		mV decade^−1^	mV decade^−1^		
Bare copper	111.1	42.1	186.2	12.91	–
Copper + Ni	124.7	48.7	177.4	5.01	61.2
Copper + Ni-G	136.2	77.3	166.8	3.79	70.6
Copper + Ni-MA	135.1	80.7	116.0	1.06	91.8
Copper + Ni-G-MA	134.8	89.3	227.8	0.93	92.8

#### EIS results

**Figure 9:**
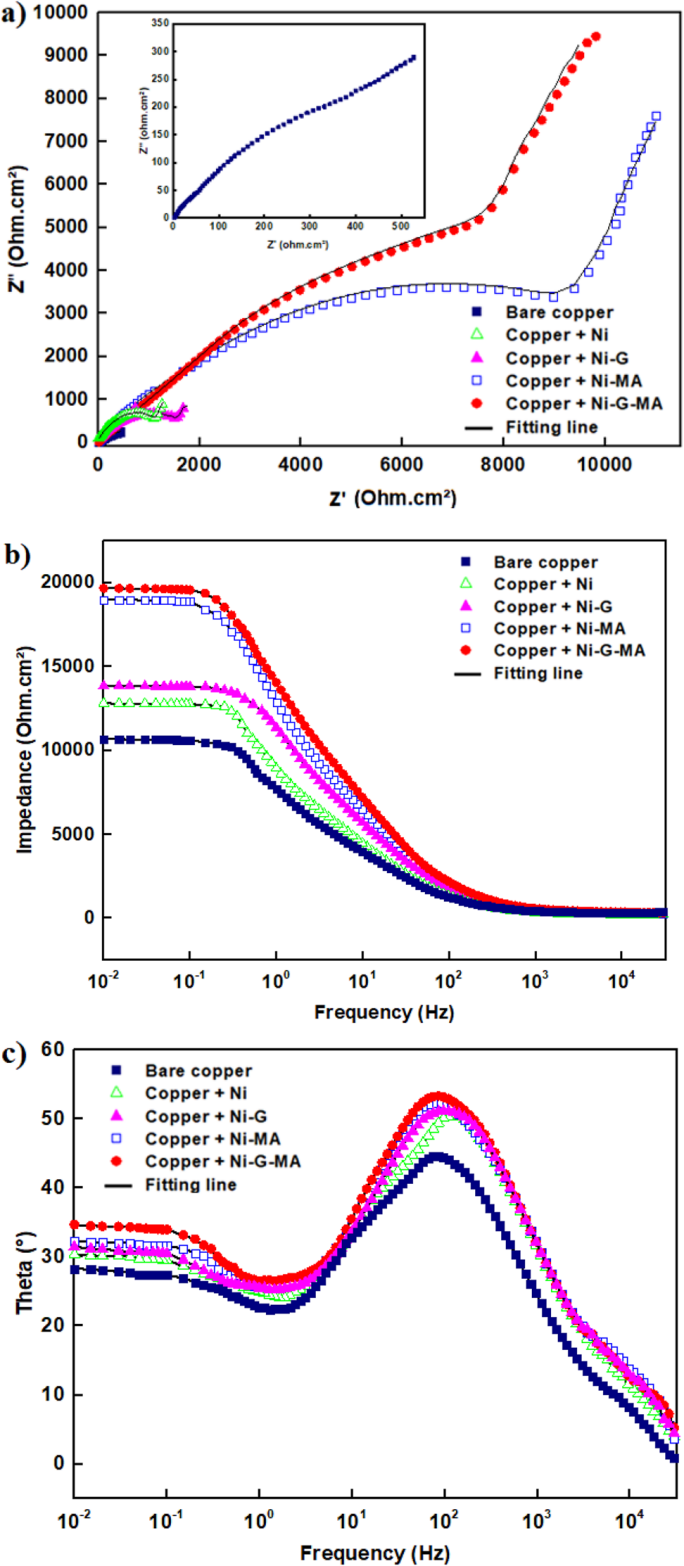
Nyquist and Bode plots of the bare and coated copper with Ni, Ni-G, Ni-MA, and Ni-G-MA in 0.5 M NaCl solution.

The Nyquist and Bode diagrams of bare and coated copper by Ni, Ni-G, Ni-MA, and Ni-G-MA in 0.5 M NaCl solution at room temperature are shown in Fig. 9. The Nyquist diagrams, Fig. 9a, exhibit a diffusion tail in the low-frequency range and a depressed capacitive semicircle in the high-frequency range. The Nyquist plots' depressed capacitive semicircle is caused by the interfacial charge transfer response^65,66^. The mass transport mechanism is responsible for the diffusion tail. It is a popular approach to measure the overall anticorrosion performance of a protective system using the low-frequency impedance modulus at 0.01 Hz. As can be seen in the Bode plots in Fig. 9b, the created SHP coated-copper has a higher impedance modulus at 0.01 Hz than Ni, Ni-G, and bare copper in 0.5 M NaCl solution. This demonstrates that the developed SHP coatings are protecting the copper substrate. The phase angle curve, Fig. 9c, displays two times constant at both low and high frequencies. The time constant that was seen in the low-frequency zone is due to the mass transport mechanism. The time constant that appeared at the high frequency was caused by the electrical double layer.

**Figure 10:**
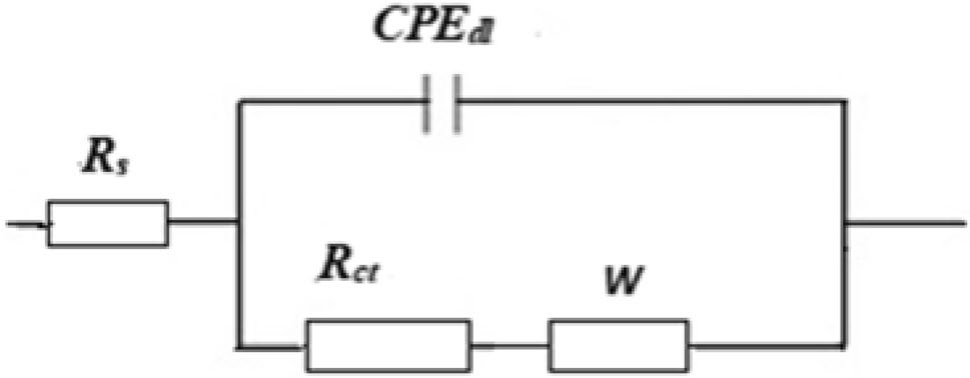
The equivalent circuit model.

The Zsimpwin program was used to estimate the impedance parameters by fitting the Nyquist diagrams to the equivalent circuit depicted in Fig. 10. The equivalent circuit includes; charge transfer resistance at copper/solution interface, R_ct_, double-layer constant phase element, CPE_dl_, which represent the non-ideal double layer capacitance at copper/solution interface, Warburg element, W, which represents the diffusion impedance behaviour at the electrode surface, and solution resistance, R_s_. The electrochemical impedance parameters of bare and coated copper are shown in Table 2. The protective efficiency is calculated using Eq. (3)^67^:

3 $$ \% {\text{P }} = \, \left[ {\left( {{\text{R}}_{{{\text{ct}}}} - {\text{ R}}_{{{\text{ct}}}}^{{\text{o}}} } \right)/{\text{ R}}_{{{\text{ct}}}} } \right] \, \times {1}00 $$

**Table 2:** Electrochemical impedance spectroscopy parameters for bare and coated copper by Ni, Ni-G, Ni-MA, and Ni-G-MA in 0.5 M NaCl solution.

Deposit	R_s_ (Ohm cm^2^)	CPE × 10^–6^ (S^n^ Ω^−1^ cm^−2^)	n	*W* (Ω^−1^ cm^−2^ S^1/2^)	R_ct_ (Ohm cm^2^)	%P
Bare copper	2.2	299	0.77	0.8	391	–
Copper + Ni	2.4	255	0.76	1.2	1217	67.9
Copper + Ni-G	2.5	207	0.74	1.4	1659	76.4
Copper + Ni-MA	4.2	114	0.74	1.5	9209	95.8
Copper + Ni-G-MA	4.4	96	0.73	1.6	10,316	96.2

R_ct_^o^ and R_ct_ are the charge transfer resistance for the bare and coated copper. The results in the table show that coated copper by SHP films provide excellent corrosion protection while coated copper by Ni and Ni-G layers shows poor corrosion protection. This result confirms the role of the SHP layer in improving the corrosion protection of the substrate. Because of its increased superhydrophobicity, Ni-G-MA coated copper has higher protective efficiency than Ni-MA. The SHP coating on the copper surface blocks active corrosion sites and reduces corrosive species diffusion into the surface. These findings match those obtained using the potentiodynamic polarization technique.

**Figure 11:**
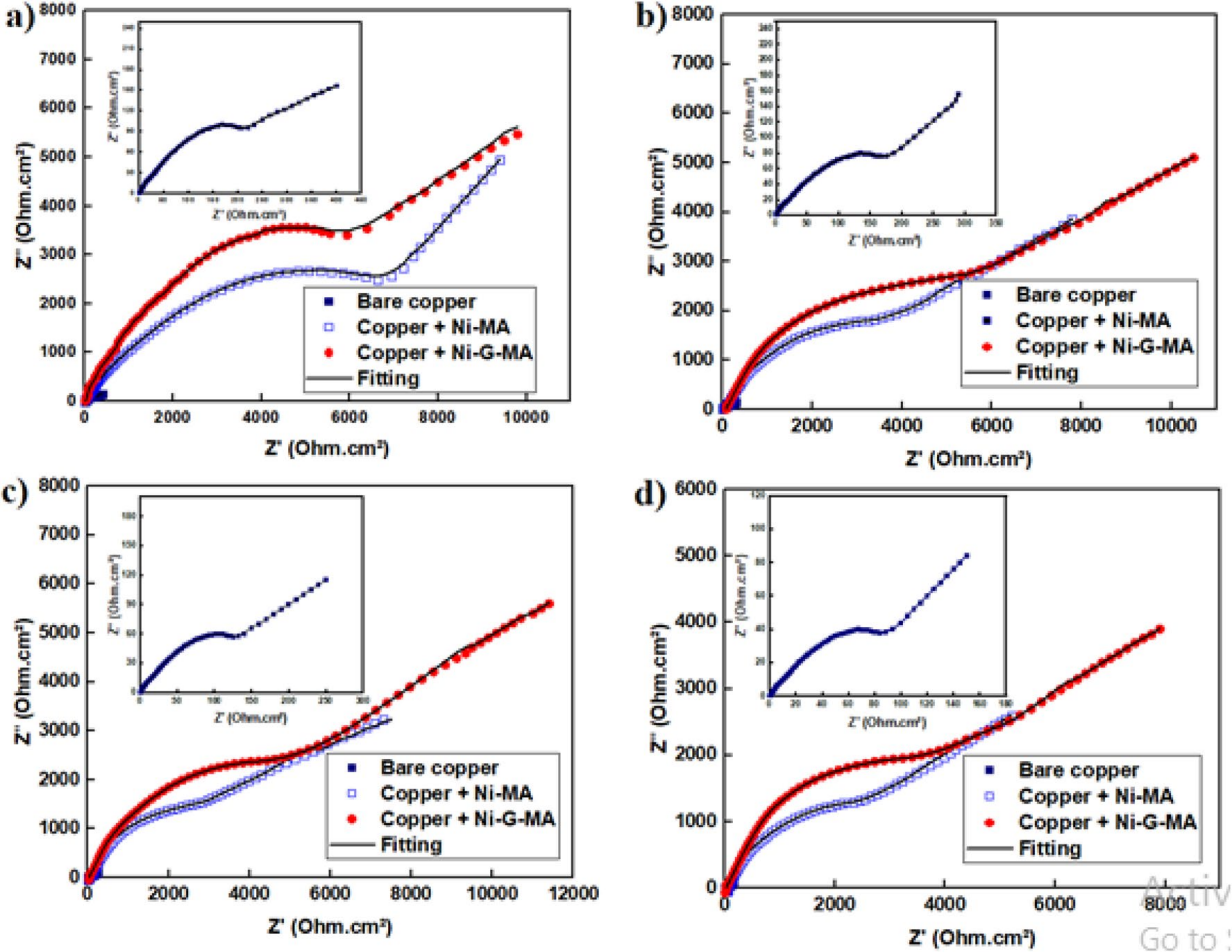
Nyquist plots of the bare and SHP coated copper immersed in 0.5 M NaCl solution for (**a**) 4 days, (**b**) 8 days, (**c**) 12 days, and (**d**) 16 days.

**Figure 12:**
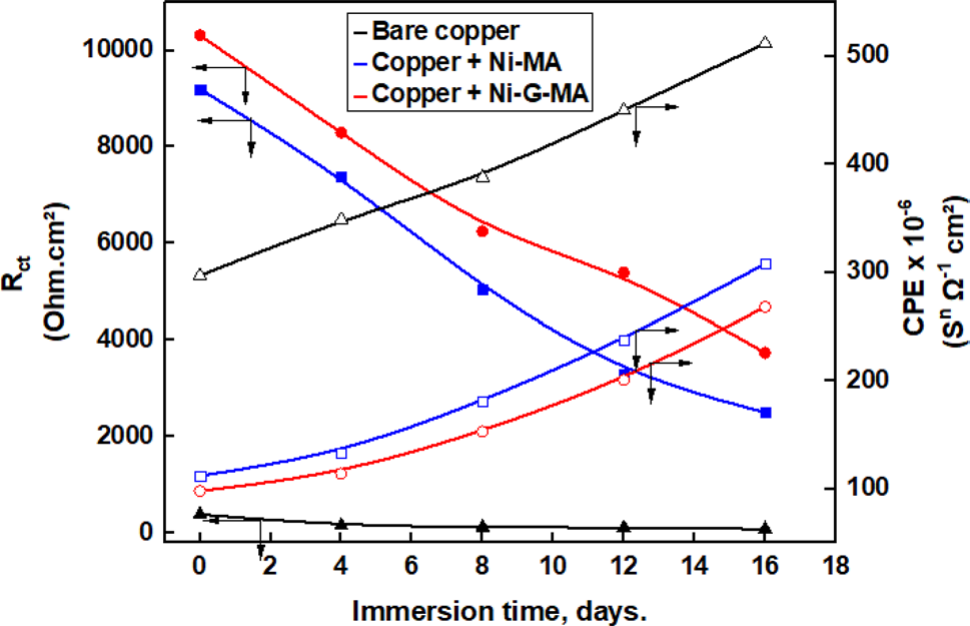
Variation of the charge transfer resistance and constant phase element of the bare and SHP coated copper as a function of immersion time.

EIS is a non-destructive method that is always used to assess a protective system's long-term corrosion resistance^41^. The variation in the charge transfer resistance value with the immersion time of the bare and SHP coated copper in 0.5 M NaCl solution was conducted for 16 days to test the corrosion resistance stability of the SHP coating^68,69^. Figure 11 shows the Nyquist plots of the uncoated and SHP coated copper after immersion for 4, 8, 12, and 16 days in 0.5 M NaCl solution. The EIS parameters were obtained by fitting the Nyquist plots with the equivalent circuit shown in Fig. 10 using the Zsimpwin program. The EIS parameters of bare and SHP coated copper are shown in Table 3. The variation of the charge transfer resistance and constant phase element of the bare and SHP coated copper as a function of immersion time is shown in Fig. 12. Evidently, the results show that during the immersion process, the charge transfer resistance decreases, and the double layer constant phase element increases for both the bare and SHP coated copper. The SHP coated copper has a protection efficiency greater than 96% after immersion in 0.5 M NaCl solution for 16 days, indicating the good durability and long-term corrosion resistance stability of the prepared SHP coats in 0.5 M NaCl solution.

**Table 3:** EIS parameters for bare and SHP coated copper immersed in 0.5 M NaCl solution for 4 days.

Immersion time	Deposit	R_s_ (Ohm cm^2^)	CPE × 10^–6^ (S^n^ Ω^−1^ cm^−2^)	n	*W* (Ω^−1^ cm^−2^ S^1/2^)	R_ct_ (Ohm cm^2^)	%P
4 days	Bare copper	1.3	348	0.80	1.0	160	–
	Copper + Ni-MA	3.3	132	0.76	1.8	7402	97.8
	Copper + Ni-G-MA	3.6	115	0.74	2.3	8301	98.1
8 days	Bare copper	1.2	389	0.78	1.7	124	–
	Copper + Ni-MA	3.1	180	0.76	2.6	5051	97.5
	Copper + Ni-G-MA	3.3	154	0.74	3.2	6252	98.0
12 days	Bare copper	1.1	451	0.78	2.5	111	–
	Copper + Ni-MA	2.9	237	0.77	3.4	3302	96.6
	Copper + Ni-G-MA	3.3	202	0.75	4.2	5401	97.9
16 days	Bare copper	1.0	512	0.76	2.9	87	–
	Copper + Ni-MA	2.8	309	0.75	3.6	2504	96.5
	Copper + Ni-G-MA	2.9	268	0.74	4.4	3740	97.7

#### Mechanism of anti-corrosion performance

Uncoated copper can cause severe corrosion because it easily adsorbs water molecules and chloride ions^17^.

**Figure 13:**
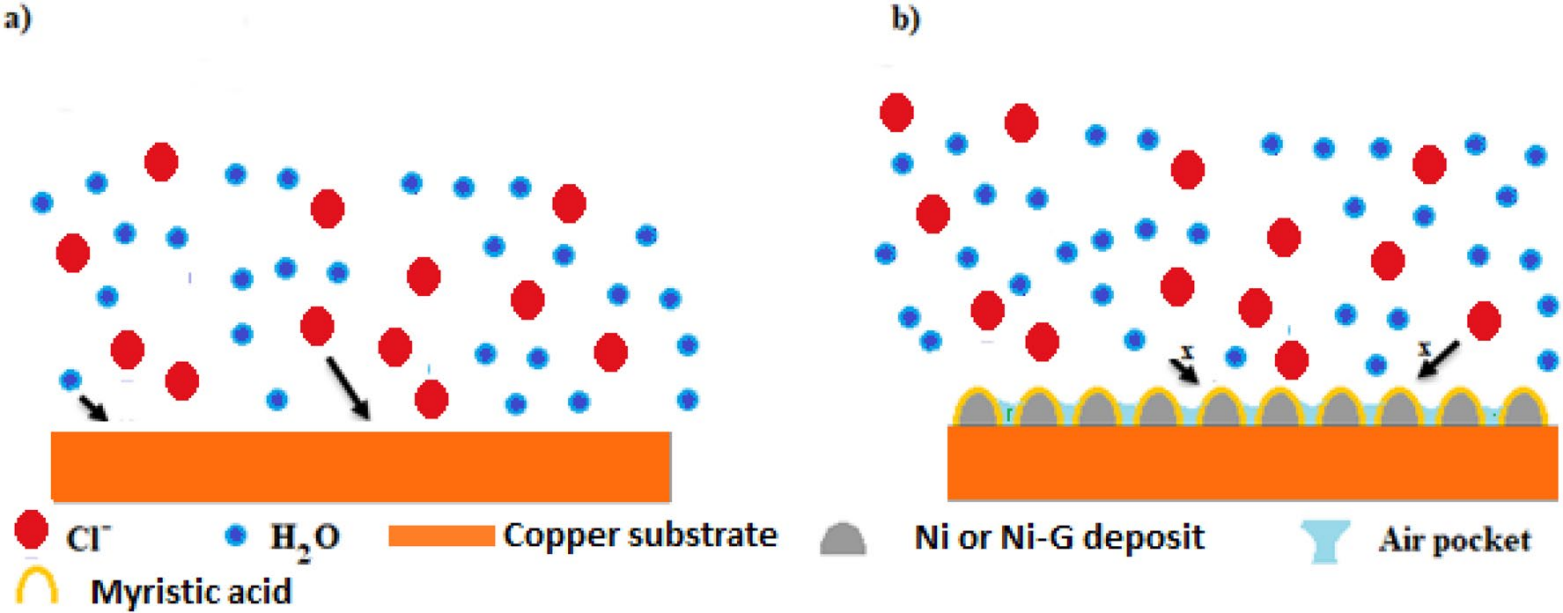
Schematic representation of the suggested mechanism for corrosion protection of the prepared SHP films.

On the other hand, the copper covered in superhydrophobic films has a nanostructure covered with a hydrophobic material. The troughs between the peaks of the rough surface are easily filled with air. Due to the obstructive effect of trapped air, aggressive ion species in corrosive environments, such as Cl^-^, may rarely assault the underlying surface^16,17,70,71^. Furthermore, it is reported that the superhydrophobic surface in neutral solutions was negatively charged. A superhydrophobic surface's negative charge led to a drop in the amount of Cl^-^ anion present close to a solid surface, increasing corrosion resistance^70^. Electronegative functional groups in graphene give it a negative zeta potential value^72–74^. Because of its higher negative surface charge and consequently lower concentration of Cl^-^ anion near solid surfaces, copper coated with Ni-G-MA exhibits better corrosion resistance than Ni-MA. Figure 13 depicts the suggested mechanism for the corrosion resistance of the generated superhydrophobic films.

## Conclusion

Two superhydrophobic films, Ni-MA and Ni-G-MA, were successfully grafted on the copper substrate. The mechanical stability of the prepared superhydrophobic films was assessed by mechanical abrasion, tape peeling, and sand impact tests. The three techniques show that the Ni-G-MA coating has higher mechanical stability than Ni-MA. The chemical stability test of the prepared superhydrophobic films shows that the Ni–G–MA film has higher chemical stability than Ni-MA in acidic and basic environments. It is obvious that adding graphene to the produced superhydrophobic coating enhances its chemical and mechanical stability. The corrosion resistance for the bare and superhydrophobic coated copper was examined by potentiodynamic polarization and EIS techniques. The results show that the superhydrophobic coating on the copper surface blocks active corrosion sites and reduces corrosive species diffusion into the copper surface. So the corrosion resistance of the superhydrophobic coated copper is high, especially if the superhydrophobic coat is doped with graphene. The long-term corrosion resistance of the prepared superhydrophobic coatings was assessed using the EIS technique, and the results show that the prepared superhydrophobic coats have high durability in 0.5 M NaCl solution.

## Data Availability

The datasets used and/or analyzed during the current study are available from the corresponding author on reasonable request.

## References

[1] Guo Z, Liu W, Su BL (2011). Superhydrophobic surfaces: From natural to biomimetic to functional. J. Colloid Interface Sci..

[2] Feng L, Li S, Li Y (2002). Super-hydrophobic surfaces: From natural to artificial super-hydrophobic surfaces: From natural to artificial. Adv. Mater..

[3] Zhang H (2018). Fabrication and drag reduction of superhydrophobic surface on steel substrates. Surf. Eng..

[4] Mohamed ME, Abd-El-Nabey BA (2022). Superhydrophobic cobalt-graphene composite for the corrosion protection of copper bipolar plates in proton exchange membrane fuel cells. J. Electrochem. Energy Convers. Storage.

[5] Pratiwi N, Zulhadjri S, Arief S, Admi A, Wellia DV (2020). Self-cleaning material based on superhydrophobic coatings through an environmentally friendly sol–gel method. J. Sol-Gel Sci. Technol..

[6] Mohamed ME, Abd-El-Nabey BA (2021). Fabrication of durable superhydrophobic/oleophilic cotton fabric for highly efficient oil/water separation. Water Sci. Technol..

[7] Su F, Yao K (2014). Facile fabrication of superhydrophobic surface with excellent mechanical abrasion and corrosion resistance on copper substrate by a novel method. ACS Appl. Mater. Interfaces.

[8] Lakshmi RV, Bharathidasan T, Bera P, Basu BJ (2012). Fabrication of superhydrophobic and oleophobic sol-gel nanocomposite coating. Surf. Coat. Technol..

[9] Lomga J (2017). Fabrication of durable and regenerable superhydrophobic coatings with excellent self-cleaning and anti-fogging properties for aluminium surfaces. J. Alloys Compd..

[10] Mohamed ME, Abd-El-Nabey BA (2022). Fabrication of a biological metal—organic framework based superhydrophobic textile fabric for efficient oil/water separation. Sci. Rep..

[11] Mohamed ME, Mahgoub FM, Ragheb DM, Abdel-Gaber AM (2021). Novel and facile method for fabrication of robust superhydrophobic film on copper surface and its chemical, mechanical, and corrosion performance. Surf. Eng..

[12] Ahmad-Kamal SA, Ritikos R, Abdul-Rahman S (2015). Wetting behaviour of carbon nitride nanostructures grown by plasma enhanced chemical vapour deposition technique. Appl. Surf. Sci..

[13] Zhang W, Wang D, Sun Z, Song J, Deng X (2021). Robust superhydrophobicity: Mechanisms and strategies. Chem. Soc. Rev..

[14] Yang Z, Liu X, Tian Y (2019). Fabrication of super-hydrophobic nickel film on copper substrate with improved corrosion inhibition by electrodeposition process. Colloids Surfaces A.

[15] Zhang B, Li Y, Hou B (2015). One-step electrodeposition fabrication of a superhydrophobic surface on an aluminum substrate with enhanced self-cleaning and anticorrosion properties. RSC Adv..

[16] Barati-Darband G, Aliofkhazraei M, Khorsand S, Sokhanvar S, Kaboli A (2020). Science and engineering of superhydrophobic surfaces: Review of corrosion resistance, chemical and mechanical stability. Arab. J. Chem..

[17] Rasitha TP, Vanithakumari SC, George RP, Philip J (2019). Template-free one-step electrodeposition method for fabrication of robust superhydrophobic coating on ferritic steel with self-cleaning ability and superior corrosion resistance. Langmuir.

[18] Qing Y, Long C, An K, Liu C (2022). Natural rosin-grafted nanoparticles for extremely-robust and eco-friendly antifouling coating with controllable liquid transport. Compos. Part B Eng..

[19] Feng L, Wang J, Shi X, Chai C (2019). Superhydrophobic copper surface with mechanical, chemical, and UV durability along with corrosion resistance and self-cleaning effect. Appl. Phys. A Mater. Sci. Process..

[20] Liu CJ, Feng XY, Li N, Luo CW, Chao ZS (2017). Super-hydrophobic Co3O4-loaded nickel foam with corrosion-resistant property prepared by combination of hydrothermal synthesis and PFAS modification. Surf. Coatings Technol..

[21] Mohamed ME, Abd-El-Nabey BA (2020). Facile and eco-friendly method for fabrication of superhydrophobic surface on copper metal. ECS J. Solid State Sci. Technol..

[22] Liu W, Xu Q, Han J, Chen X, Min Y (2016). A novel combination approach for the preparation of superhydrophobic surface on copper and the consequent corrosion resistance. Corros. Sci..

[23] Wang P, Zhang D, Qiu R, Wan Y, Wu J (2014). Green approach to fabrication of a super-hydrophobic film on copper and the consequent corrosion resistance. Corros. Sci..

[24] Tsai DC, Hwang WS (2012). Numerical simulation of the solidification processes of copper during vacuum continuous casting. J. Cryst. Growth.

[25] Fang R (2020). Atomic insight into the solidification of Cu melt confined in graphene nanoslits. J. Cryst. Growth.

[26] Liu Y (2015). Corrosion inhibition of biomimetic super-hydrophobic electrodeposition coatings on copper substrate. Corros. Sci..

[27] Huang Y, Sarkar DK, Gallant D, Chen XG (2013). Corrosion resistance properties of superhydrophobic copper surfaces fabricated by one-step electrochemical modification process. Appl. Surf. Sci..

[28] Yu D, Tian J (2014). Superhydrophobicity: Is it really better than hydrophobicity on anti-corrosion?. Colloids Surfaces A Physicochem. Eng. Asp..

[29] Lv Y, Liu M (2019). Corrosion and fouling behaviours of copper-based superhydrophobic coating. Surf. Eng..

[30] Khorsand S, Raeissi K, Ashrafizadeh F (2014). Corrosion resistance and long-term durability of super-hydrophobic nickel film prepared by electrodeposition process. Appl. Surf. Sci..

[31] Mohamed ME, Ezzat A, Gaber AMA (2022). Fabrication of eco—friendly graphene—based superhydrophobic coating on steel substrate and its corrosion resistance, chemical and mechanical stability. Sci. Rep..

[32] Montemor MF (2014). Functional and smart coatings for corrosion protection: A review of recent advances. Surf. Coat. Technol..

[33] Chen-Yang YW, Yang HC, Li GJ, Li YK (2005). Thermal and anticorrosive properties of polyurethane/clay nanocomposites. J. Polym. Res..

[34] Mohamed ME, Abd-El-Nabey BA (2022). Corrosion performance of a steel surface modified by a robust graphene-based superhydrophobic film with hierarchical roughness. J. Mater. Sci..

[35] Compton OC, Nguyen ST (2010). Graphene oxide, highly reduced graphene oxide, and graphene: Versatile building blocks for carbon-based materials. Small.

[36] Yu P, Lowe SE, Simon GP, Zhong YL (2015). Electrochemical exfoliation of graphite and production of functional graphene. Curr. Opin. Colloid Interface Sci..

[37] Hu D, Li Y, Weng Y, Peng H, Zeng J (2022). Fabrication of sustainable and durable superwetting cotton fabrics with plant polyphenol for on-demand oil/water separation. Ind. Crop. Prod..

[38] Cassie ABD, Baxter S (1944). Wettability of porous surfaces. Trans. Faraday Soc..

[39] Manoj TP (2020). A simple, rapid and single step method for fabricating superhydrophobic titanium surfaces with improved water bouncing and self cleaning properties. Appl. Surf. Sci..

[40] Abd-El-Nabey BA, Ashour M, Aly A, Mohamed M (2022). Fabrication of robust superhydrophobic nickel films on steel surface with high corrosion resistance, mechanical and chemical stability. J. Eng. Mater. Technol..

[41] Jiang D (2019). A novel coating system with self-reparable slippery surface and active corrosion inhibition for reliable protection of Mg alloy. Chem. Eng. J..

[42] Valencia C (2018). Synthesis and application of scaffolds of chitosan-graphene oxide by the freeze-drying method for tissue regeneration. Molecules.

[43] Jena G, Thinaharan C, George RP, Philip J (2020). Robust nickel-reduced graphene oxide-myristic acid superhydrophobic coating on carbon steel using electrochemical codeposition and its corrosion resistance. Surf. Coat. Technol..

[44] Zhu J (2016). Simple and green fabrication of a superhydrophobic surface by one-step immersion for continuous oil/water separation. J. Phys. Chem. A.

[45] Khairuddin E (2016). FTIR studies on the effect of concentration of polyethylene glycol on polimerization of Shellac. J. Phys. Conf. Ser..

[46] She Z (2013). Researching the fabrication of anticorrosion superhydrophobic surface on magnesium alloy and its mechanical stability and durability. Chem. Eng. J..

[47] Zhang X, Guo Y, Zhang Z, Zhang P (2013). Self-cleaning superhydrophobic surface based on titanium dioxide nanowires combined with polydimethylsiloxane. Appl. Surf. Sci..

[48] Tan C, Li Q, Cai P, Yang N, Xi Z (2015). Fabrication of color-controllable superhydrophobic copper compound coating with decoration performance. Appl. Surf. Sci..

[49] Peng C, Chen Z, Tiwari MK (2018). All-organic superhydrophobic coatings with mechanochemical robustness and liquid impalement resistance. Nat. Mater..

[50] Cao C (2020). Sprayable superhydrophobic coating with high processibility and rapid damage-healing nature. Chem. Eng. J..

[51] Li L (2021). One-step spraying method to construct superhydrophobic magnesium surface with extraordinary robustness and multi-functions. J. Magnes. Alloy.

[52] Zulfiqar U (2017). Durable and self-healing superhydrophobic surfaces for building materials. Mater. Lett..

[53] Xu S, Wang Q, Wang N, Zheng X (2019). Fabrication of superhydrophobic green surfaces with good self-cleaning, chemical stability and anti-corrosion properties. J. Mater. Sci..

[54] Wang J, Wu Y, Cao Y, Li G, Liao Y (2020). Influence of surface roughness on contact angle hysteresis and spreading work. Colloid Polym. Sci..

[55] Guo M, Kang Z, Li W, Zhang J (2014). A facile approach to fabricate a stable superhydrophobic film with switchable water adhesion on titanium surface. Surf. Coat. Technol..

[56] Ma L (2021). Preparation of a superhydrophobic TiN/PTFE composite film toward self-cleaning and corrosion protection applications. J. Mater. Sci..

[57] Du C, He X, Tian F, Bai X, Yuan C (2019). Preparation of superhydrophobic steel surfaces with chemical stability and corrosion. Coatings.

[58] Flitt HJ, Schweinsberg DP (2005). Evaluation of corrosion rate from polarisation curves not exhibiting a Tafel region. Corros. Sci..

[59] McCafferty E (2005). Validation of corrosion rates measured by the Tafel extrapolation method. Corros. Sci..

[60] Fetouh HA, Abd-El-Nabey B, Goher YM, Karam MS (2018). An electrochemical investigation in the anticorrosive properties of silver nanoparticles for the acidic corrosion of aluminium. J. Electrochem..

[61] Ou J (2012). Corrosion behavior of superhydrophobic surfaces of Ti alloys in NaCl solutions. Appl. Surf. Sci..

[62] Ramesh S, Rajeswari S, Maruthamuthu S (2003). Effect of inhibitors and biocide on corrosion control of mild steel in natural aqueous environment. Mater. Lett..

[63] Devi NR (2020). Extracts of leaves as corrosion inhibitors—an overview and corrosion inhibition by an aqueous extract of henna leaves (*Lawsonia inermis*). Int. J. Corros. Scale Inhib..

[64] Bahlakeh G, Dehghani A, Ramezanzadeh B, Ramezanzadeh M (2019). Highly effective mild steel corrosion inhibition in 1 M HCl solution by novel green aqueous Mustard seed extract: Experimental, electronic-scale DFT and atomic-scale MC/MD explorations. J. Mol. Liq..

[65] Ghiamati-Yazdi E, Ghahfarokhi ZS, Bagherzadeh M (2017). Protection of carbon steel corrosion in 3.5% NaCl medium by aryldiazonium grafted graphene coatings. New J. Chem..

[66] Wan S, Miao CH, Wang RM, Zhang ZF, Dong ZH (2019). Enhanced corrosion resistance of copper by synergetic effects of silica and BTA codoped in polypyrrole film. Prog. Org. Coat..

[67] Abd-El-nabey BA, Goher YM, Fetouh HA, Karam MS (2015). Anticorrosive properties of chitosan for the acid corrosion of aluminium. Port. Electrochim. Acta.

[68] Abbasi S, Nouri M, Sabour Rouhaghdam A (2019). A novel combined method for fabrication of stable corrosion resistance superhydrophobic surface on Al alloy. Corros. Sci..

[69] Kuang J, Ba Z, Li Z, Wang Z, Qiu J (2020). The study on corrosion resistance of superhydrophobic coatings on magnesium. Appl. Surf. Sci..

[70] Ou J, Chen X (2019). Corrosion resistance of phytic acid/Ce (III) nanocomposite coating with superhydrophobicity on magnesium. J. Alloys Compd..

[71] Chen X, Wang P, Zhang D, Ou J (2022). Effect of surface nanostructure on enhanced atmospheric corrosion resistance of a superhydrophobic surface. Colloids Surfaces A Physicochem. Eng. Asp..

[72] Krishnamoorthy K, Veerapandian M, Yun K, Kim SJ (2013). The chemical and structural analysis of graphene oxide with different degrees of oxidation. Carbon N. Y..

[73] Smith RJ, Lotya M, Coleman JN (2010). The importance of repulsive potential barriers for the dispersion of graphene using surfactants. New J. Phys..

[74] Baskoro F (2018). Graphene oxide-cation interaction: Inter-layer spacing and zeta potential changes in response to various salt solutions. J. Memb. Sci..

